# The School of Medicine for Women

**Published:** 1889-07-06

**Authors:** 


### The School of Medicine for Women.

The presence of the Marchioness of Dufferin and Ava at the School of Medicine for Women, on the 25th ult., caused the lecture-hall, and even the old-fashioned garden of the house in Handel-street, to be filled with an eager crowd. Her ladyship, who was accompanied by her daughters, the Ladies Helen and Hermione Blackwood, distributed the prizes and certificates to the successful students, and among those present were Mrs. Garrett Anderson, M.D., the dean of the school, Dr. Annie M'Call, Dr. Elizabeth Blackwell, Sir Guyer Hunter, Dr. Samuel West, of the Royal Free Hospital, General Pritchard, and many other friends of the school. A very interested spectator was Mrs. Carmichael, who, as far back as 1872, began with Lady Napier, of Ettrick, to plead for the introduction of medical women into India, and everybody was asking the name of the Burmese lady and gentleman whose dress and diamonds attracted general attention. They were finally discovered to be Mr. and Mrs. M. Hla Oung, members of the really enlightened class of natives. Mrs. Oung's dress consisted of a short tight robe of rose-pink silk, with a scarf of a lighter colour, and a wreath of flowers in her hair. The statement made by Mrs. Garrett Anderson showed that the school is increasing in numbers, twenty-two new students having entered last October, while Mr. Stansfeld, the honorary treasurer, proved that its financial condition was satisfactory. Many people were surprised at the youth of the students, who, for the most part, are just like any other happy, bright-looking girls. After they had received their prizes, Lady Dufferin addressed a few words to them, with special reference to those who had made India their goal. She warned them that they had taken up a profession that involved hard work and unselfish devotion to duty; that for them there would be no ease or self-indulgence. In India especially they would have to contend with a trying climate, imperfect hospital accommodation, and inefficient nursing; but, at the same time, they would have great opportunities for working and for doing good. They would need all their learning, all their training, all the calm and self-control, all the presence of mind they could command; but Lady Dufferin assumed that they had not taken up their profession lightly, and would qualify themselves to be worthy members of it. Sir Guyer Hunter, in proposing a vote of thanks to the marchioness for her presence, alluded to the great work she had done in establishing the fund that bears her name, and Dr. Samuel West expressed his belief that it was due partly to her influence that the Marquis of Dufferin had consented to become president of the Royal Free Hospital. Lady Dufferin briefly expressed the pleasure it gave her to be present, and then the company wandered into the garden, where the students proved that the study of medicine had not made them forget the humbler art of serving afternoon tea.

